# Phenomenology of the stream of thought: dissociable dynamic dimensions revealed through experience sampling

**DOI:** 10.1093/nc/niag017

**Published:** 2026-05-18

**Authors:** Sneha K S Sheth, Mike Doswell, Kalina Christoff Hadjiilieva, Rebecca M Todd, Evan Thompson, Lawrence M Ward

**Affiliations:** University of British Columbia, Department of Psychology, 2136 West Mall, Vancouver, BC V6T 1Z4, Canada; University of British Columbia, Department of Psychology, 2136 West Mall, Vancouver, BC V6T 1Z4, Canada; University of British Columbia, Department of Psychology, 2136 West Mall, Vancouver, BC V6T 1Z4, Canada; University of British Columbia, Department of Psychology, 2136 West Mall, Vancouver, BC V6T 1Z4, Canada; University of British Columbia, Djavad Mowafaghian Centre for Brain Health, 2215 Wesbrook Mall, Vancouver, BC V6T 1Z3, Canada; University of British Columbia, Department of Philosophy, 1866 Main Mall E370, Vancouver, BC V6T 1Y1, Canada; University of British Columbia, Department of Psychology, 2136 West Mall, Vancouver, BC V6T 1Z4, Canada; University of British Columbia, Djavad Mowafaghian Centre for Brain Health, 2215 Wesbrook Mall, Vancouver, BC V6T 1Z3, Canada

**Keywords:** phenomenology of thought, stream of thought, thought dynamics, experience sampling, first-person experience, neurophenomenology

## Abstract

The pursuit of a scientific theory of consciousness has gained momentum in neuroscience over the past 25 years. While much research has centered on perceptual consciousness, the dynamic experience of the stream of thought, proposed by William James over a century ago, has remained relatively underexplored. The Dynamic Framework of Thought (DFT) introduced a taxonomy of thought dynamics, emphasizing their role in shaping conscious experiences. The present study explores the introspective accessibility and distinctiveness of two dynamic dimensions of thought, freely moving and deliberately directed, derived from the taxonomy of the DFT. To investigate these dynamics, four experiments were conducted, including laboratory-based and online experiments as well as an fMRI-based experiment, to assess the consistency of the relationship between these thought dynamics across various experimental contexts. In all experiments, participants reported the dynamics of their thoughts during a probed resting period in which they sat quietly with eyes open, viewing a blank screen. Using a mixed-methods approach combining qualitative and quantitative assessments, this study suggests that individuals can have some introspective access to their thought dynamics. Freely moving and deliberately directed thoughts were distinguishable and negatively correlated but not anticorrelated across participants and experimental contexts. These findings suggest that the stream of thought can be characterized by at least two distinct dynamic dimensions—freely moving and deliberately directed—offering valuable insights for future neurophenomenological research aimed at bridging first-person reports of thought dynamics with third-person data on brain processes.

## Introduction

The search for a scientific theory of consciousness has gained significant momentum over the past 25 years, particularly in neuroscience (Ward 2011, Seth and Bayne 2022). However, this work has mostly focused on the perceptual consciousness of the outside world (Pascual-Leone and Walsh 2001, Dehaene et al. 2003, Lamme 2003, Hameroff and Penrose 2014, Bayne et al. 2016, Tononi et al. 2016, Cleeremans et al. 2025). Over a century ago, William James called attention to a different facet of consciousness, its dynamics, which he called the ‘stream of thought’ (James 1890). Until recently, however, it has remained a relatively underexplored aspect of consciousness (Lux et al. 2022, Kucyi et al. 2023, Su et al. 2024, Rasmussen et al. 2024a, Rasmussen et al. 2024b, Feng et al. 2025, Garg et al. 2025, Tripathi et al. 2025). The stream of thought is a metaphor for the idea that much of our conscious waking life comprises thoughts that frequently move in time from one mental state to another. In this paper we explore the dynamic experience of the stream of thought from a particular theoretical viewpoint, the Dynamic Framework of Thought (DFT) (Christoff et al. 2016, Girn et al. 2020).

The DFT proposes a taxonomy of thought defined by its dynamics rather than by its more or less static content. Importantly, these dynamics are influenced by a variety of constraints that shape the movement of thoughts between different mental states. According to the DFT, constraints on thought can be implemented in two ways. One type of constraint, termed ‘deliberate constraint,’ involves flexible and deliberate control, in which cognitive effort is utilized to focus on specific tasks or contents. For instance, we can deliberately direct our attention to a boring lecture and redirect our thoughts back to it whenever they wander. The second type of constraint, termed ‘automatic constraint,’ may operate outside of cognitive control and restrict attention to a particular set of information. Affective and sensory salience are examples of automatic constraints. Despite our best intentions, we might still struggle to divert our attention from a distracting fly buzzing in a quiet library or from emotionally absorbing concerns. Spontaneous thought, characterized as ‘freely moving’ thought within DFT, is a sequence of mental states with relatively few constraints controlling transitions between them (Appendix A).

Since the introduction of DFT, several studies have begun to incorporate measures of dynamics of thought into their empirical work (Mills et al. 2018, 2021, Smith et al. 2018, Alperin et al. 2021, Kam et al. 2021, O’Neill et al. 2021, Raffaelli et al. 2021, Andrews-Hanna et al. 2022, Smith et al. 2022, Poulos et al. 2023). For example, Mills et al. (2018) found that freely moving thought and task-unrelated thought shared only a weak correlation. O’Neill et al. (2021) found that freely moving thought, task-unrelated thought, and intentionality are not redundant with one another. Smith et al. (2022) showed that accounting for thought constraint produced novel results when re-examining the effect of motivation on intentional and unintentional task-unrelated thought.

In the present study, we explored two dynamic dimensions—freely moving and deliberately directed—derived from DFT (Christoff et al. 2016). We use the term ‘freely moving thought’ in this paper to refer to spontaneous thought as defined in the DFT (Mills et al. 2018, 2021, Smith et al. 2018, Alperin et al. 2021, Kam et al. 2021, O’Neill et al. 2021, Raffaelli et al. 2021, Andrews-Hanna et al. 2022, Poulos et al. 2023). We characterize it as thought that proceeds in the absence of strong constraints, whether deliberate or automatic, and requires little cognitive effort. It seems to flow along from state to state, or between topics, ‘of its own accord.’ On the other hand, we characterize ‘deliberately directed thought’ as thought that requires cognitive effort and goal-directed processes. Whereas these two dynamic dimensions seem somewhat conceptually opposed, our findings suggest that they represent distinct experiences within the stream of thought, not merely the opposite ends of a single continuum. For example, a person could deliberately focus on planning their upcoming weekend trip (high directedness) while their thoughts flow freely among different possibilities—places to visit, the possibility of inviting a friend, what to pack, a memory of a previous trip, and how to get there (high free movement).

We note that directedness, as used here, refers to the experience of actively steering or controlling the course of one’s thought toward a goal. This is distinct from intentionality (Seli et al. 2016), which refers to whether one intended to begin thinking about something. A person could intentionally decide to let their thoughts wander (high intentionality, low directedness), or could unintentionally find themselves focused on a problem (low intentionality, high directedness). The relationship between directedness and intentionality has been examined empirically by O’Neill et al. (2021) and Smith et al. (2022), who found these to be dissociable dimensions.

We explored freely moving and deliberately directed dimensions because they are likely to be introspectively accessible. In contrast, automatic constraints on thought by definition operate mostly outside of cognitive control and are less likely to be available for conscious report (Christoff et al. 2016). We focused on these two dimensions to maintain consistency between our behavioral experiments and neuroimaging experiments (e.g. Sheth et al. 2025), as one of the initial objectives was to test the feasibility of our study design for the fMRI scanner, which posed the limitation of allowing only two questions.

Four experiments were designed to answer the following questions: (i) Can we use experience sampling to obtain informative first-person data on freely moving and deliberately directed thought? (ii) What is the relationship between freely moving thought and deliberately directed thought? (iii) What can we learn from qualitative reports of participants’ experiences with reporting these dynamics? We hypothesized that freely moving and deliberately directed thought are empirically dissociable dynamic dimensions, consistent with their theoretical distinction in DFT. We used experience sampling because it is a well-established method for capturing moment-to-moment experience and has been effective in prior studies of thought dynamics (Csikszentmihalyi and Larson 1987, Christoff et al. 2009a, Stawarczyk et al. 2011, Timmermans and Cleeremans 2015, Smith et al. 2018, Mills et al. 2021, Cleeremans et al. 2025).

Experiments 1 and 2 were laboratory-based experiments with minor variations, conducted in part to check feasibility for the fMRI-based Experiment 3 (described in detail in Sheth et al. 2025), whose behavioral data were incorporated here to assess context sensitivity. Experiment 4 was conducted online and additionally required qualitative responses to help determine whether participants based their ratings on the felt experience of thought dynamics or on some other process, such as inference from the amount of change in thought content.

Because previous studies have defined mind-wandering primarily through content-based dimensions such as task-unrelatedness and perceptual decoupling (Smallwood and Schooler 2006, Mason et al. 2007, Stawarczyk et al. 2011, Kam et al. 2012, Mills et al. 2018), whereas DFT defines it as a type of freely moving thought whose central feature lies in the way it moves (Christoff et al. 2016, Girn et al. 2020), we also examined whether dynamic dimensions were dissociable from content-based dimensions such as perceptual (de)coupling in our qualitative data.

In all of the present experiments, participants were instructed to allow their thoughts to unfold naturally, and they were periodically asked to rate the dynamics of the thoughts they had experienced in the few seconds just preceding the probe, on a scale of 1 to 6 for each of ‘freely moving’ and ‘deliberately directed.’ Single-item scales were used to minimize probe duration and participant burden. These two dynamics are not intended to be an exhaustive taxonomy of thought, but rather to represent two tractable experiential dimensions predicted by the DFT that can guide future neurophenomenological studies linking first-person reports to brain processes (Lutz and Thompson 2003, Thompson et al. 2005, Kam et al. 2021, Cleeremans et al. 2025, Sanchez Sanchez et al. 2025, Sheth et al. 2025).

## Methods

Informed consent was obtained from all participants. All research protocols described here were approved by the Behavioral Research Ethics Board at the University of British Columbia in accordance with the Declaration of Helsinki.

### Participants

A total of 900 University of British Columbia undergraduate students (708 after exclusion) participated in all our experiments for course credit or monetary compensation. The breakdown of the sample size in each experiment, along with the relevant exclusion criteria, is described below.

#### Experiment 1: Laboratory-based

A total of 70 undergraduate students at the University of British Columbia (60 after exclusion) participated in our experiment in exchange for course credit (*M*age = 20.02 (range: 18–37), *SD* = 2.79; 72% female, 28% male). Of the 10 participants that were excluded, two were excluded because they did not participate in the experiment, seven were excluded because they missed more than 30% of the probes in the experiment, and one was excluded because they pressed the same key in all the trials for the deliberately directed question.

#### Experiment 2: Laboratory-based

A total of 91 participants (82 after exclusion) participated in exchange for monetary compensation (*M*age = 25.88 (range: 20–35), *SD* = 3.88; 72% female, 28% male). Of the nine excluded participants, four were excluded because they did not attend the experiment, four because they missed more than 30% of the probes in the experiment, and one because they had a standard deviation of 0 on the freely moving question (i.e. they pressed the same key throughout the experiment).

#### Experiment 3: fMRI scanner

A total of 24 participants participated in exchange for monetary compensation (*M*age = 27.4 (range: 22–35), *SD* = 4.14; 63% female, 37% male). None of the participants were excluded from the experiment. These participants were selected from the Experiment 2 sample based on availability.

#### Experiment 4: Online

A total of 715 undergraduate students (542 after exclusion) participated for course credit (*M*age = 20.41, range: 18–47, *SD* = 2.55; 72% female, 25% male, 3% non-binary). Exclusions: 34 did not finish, five had empty data files, 96 completed fewer than four explanation trials, 1 had average explanation length under two characters, 34 missed more than 30% of rating trials, and 3 had a standard deviation of 0 on either question.

## Procedure

### Basic procedure

We employed a real-time experience-sampling method (Christoff et al. 2009a) using a computer-based experiment in which participants were shown a blank, white, computer screen and instructed to let their thoughts unfold naturally while keeping their eyes open. Approximately once every minute (pseudo-randomly scheduled in a range of 40–60 seconds, mean: 50 seconds; see Appendix B, Fig. B1), participants were probed on their train of thought immediately prior to the probe using two questions: 1) Was your mind moving freely? 2) Were you actively directing your thoughts? They provided ratings on a scale of 1 (not at all) to 6 (very much) for each question. They had 4 seconds to answer each question. The question order was kept consistent across all probes and experiments to minimize cognitive load. Prior to the experiment, participants received detailed instructions (Appendix A) with examples and participated in a practice session with feedback. The experiment duration was 60–90 minutes, with periodic breaks to mitigate fatigue. By allowing thoughts to unfold in a minimally constrained setting, we aimed to create conditions in which thought dynamics could be observed with minimal interference from external task demands. This basic procedure was common to all four experiments; experiment-specific variations are described below.

#### Experiment 1: Laboratory-based

This experiment was conducted in a laboratory in the Douglas T. Kenny Building at the University of British Columbia. The experiment consisted of six blocks, with 12 probes per block, amounting to 72 probes in total. A third question about automatically constrained thought was included but excluded from analysis to maintain consistency with subsequent experiments.

#### Experiment 2: Laboratory-based

This experiment was also conducted in the same laboratory as Experiment 1. The experiment consisted of three blocks of 12 probes per block and one block of six probes, amounting to 42 probes in total.

#### Experiment 3: fMRI scanner

Behavioral data from a brain imaging experiment (Sheth et al. 2025) were incorporated here. The experiment used the same basic procedure inside the fMRI scanner, consisting of six blocks, with 12 probes per block, amounting to 72 probes in total.

#### Experiment 4: Online

Participants were given a web link and instructed to sit in a quiet, distraction-free environment. To prevent duplicate responses, unique session IDs and system checks were employed. The experiment was implemented using Qualtrics, Pavlovia, and PsychoPy. The experiment consisted of six blocks, with participants probed between 45–47 times in total. After every ~ 4 probes, participants were given 30 seconds to type a brief explanation of their just preceding rating into a text box on the screen. This resulted in 6316 explanations across the 542 participants included in this experiment.

### Qualitative coding procedure

The goal was to determine the metacognitive basis for participants’ ratings—specifically, whether they relied on the felt phenomenology of thought dynamics (dynamic-related) or inferred dynamics from changes in thought content (content-related). NVIVO (Lumivero, Denver, CO, USA) was used to generate a frequency-ranked word list from all explanations. Two coders independently classified each word as dynamic-related (e.g. flowing, drifting, stuck, fixated) or not, achieving high agreement (κ > .80). Discrepancies were resolved through discussion. A custom R routine then searched each explanation for the presence of at least one dynamic-related word; if present, the explanation was classified as dynamic-related; otherwise, as content-related (though this binary classification may not capture all possible metacognitive strategies; see Discussion). The coding scheme was designed to detect explicit dynamic language, not the full presence or absence of phenomenological access. Whereas inter-rater agreement supports the reliability of this coding process, it does not by itself guarantee validity. The same procedure was applied to classify explanations into additional categories, including mind-blanking, perceptual coupling, task interference, and meditation (see Appendix D.1 for full category definitions and Appendix C for examples of participant explanations in each category).

## Results

In what follows we use the terms ‘freeness’ and ‘directedness’ to refer to the two scales inclusive of all raw responses from 1 to 6 on both scales. In contrast, we use the terms ‘freely moving’ and ‘deliberately directed’ to refer to thoughts that were rated as a 4, 5, or 6 on the freeness and directedness scale, respectively. Likewise, thoughts that were rated 1, 2, or 3 on the freeness and directedness scales are referred to as ‘not freely moving’ and ‘not deliberately directed,’ respectively. This binarization (ratings 1–3 versus 4–6) was used only for the descriptive purposes (see *Co-occurrence of freeness and directedness*); all primary inferential analyses used all numerical ratings.

### Descriptive statistics and relative frequency

Across all 708 participants and 32 381 probe responses, the mean freeness rating was 4.13 (*SD* = 0.91) and the mean directedness rating was 2.84 (*SD* = 0.84). Freely moving thoughts (ratings 4–6) were approximately twice as frequent as deliberately directed thoughts (21 046 versus 10 675 probe responses), suggesting that freely moving thoughts predominate in the probed resting-state stream of thought.

### The relationship between freeness and directedness across various experimental contexts

**Figure 1:**
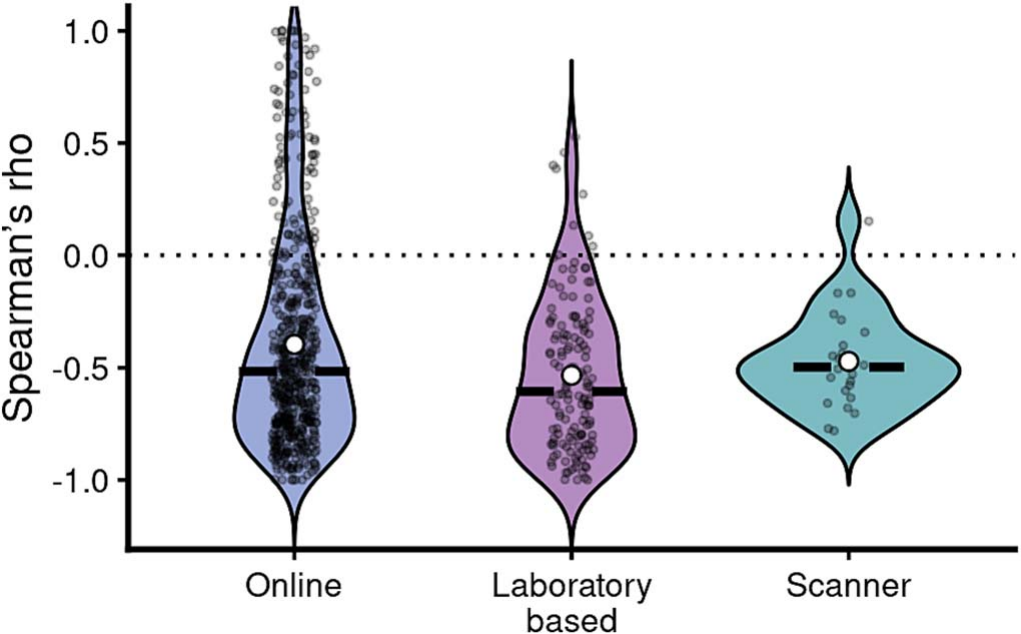
Correlation between freeness and directedness across contexts.

To test whether the two laboratory-based datasets differed before combining them, we fitted linear mixed-effects models with dataset (Experiment 1 versus Experiment 2) as a fixed effect and participant as a random intercept to account for the nested structure of the data (multiple probe responses per participant). Separate models were fitted for freeness and directedness ratings. These analyses revealed no significant effect of dataset on freeness ratings (β = −0.02, *SE* = 0.14, *t* = −0.14, *P* = .89) or directedness ratings (β = 0.11, *SE* = 0.14, *t* = 0.76, *P* = .45). The two laboratory-based datasets were therefore combined for subsequent analyses. For each of the three remaining datasets, we computed Spearman’s *rho* between freeness and directedness ratings for each individual using their raw scale responses on the freeness and directedness dimensions across all probes. We then averaged across individuals within each of the three groups for group-level correlations between freeness and directedness in each context. We found that the median Spearman’s *rho* (Fig. 1) was −0.52, −0.61, and − 0.50, and the mean Spearman’s *rho* was −0.41, −0.53, and − 0.47 in the online, laboratory-based, and scanner versions respectively. This suggests that freeness and directedness have a consistent, negative correlation that does not meaningfully differ across various experimental contexts.

Violin plots of intra-individual correlations between freeness and directedness across three experimental contexts: online (*N* = 542), laboratory-based (*N* = 142), and scanner (*N* = 24). The circle represents the median and the horizontal line represents the mean.

### The overall relationship between freeness and directedness

Across all 708 participants, the median and mean Spearman ranked correlations were − 0.53 and − 0.43 (*SD* = 0.44), respectively. To verify that the negative relationship between freeness and directedness was not an artifact of treating multiple probes from each individual as independent data, we fit a linear mixed effects model predicting freeness from directedness, with random intercepts and random slopes for directedness by participant. Consistent with our findings, the model showed a significant negative fixed effect of directedness (β = −0.42, *SE* = 0.02, *t* = −25.73, *P* < .001), suggesting that higher directedness was associated with lower freeness even after correcting for repeated measures from the same participant. Whereas 19% of participants had correlations near zero or positive (−0.1 ≤ *ρ* ≤ 1), 81% showed a negative correlation (−1 ≤ *ρ* < −0.1).

### Co-occurrence of freeness and directedness

To further characterize dissociability, we examined co-occurrence using a binarized 2 × 2 matrix (Table 1). Table 1 provides probe-level co-occurrence information (e.g. 12% of individual responses rated as both freely moving and deliberately directed) that is not visible in the person-level violin plots. If the two dimensions were entirely redundant, the co-occurrence cells would contain substantially fewer responses, after allowing for measurement noise. Instead, we observed that out of 32 381 probe responses, 12% (3782) were categorized as both freely moving and deliberately directed, and 14% (4442) as both not freely moving and not directed (Table 1).

**Table 1:** Co-occurrence of freely moving and deliberately directed thought ratings.

	**Freely moving**	**Not freely moving**	**Total**
**Not deliberately directed**	53% [17264]	14% [4442]	67% [21706]
**Deliberately directed**	12% [3782]	21% [6893]	33% [10675]
**Total**	65% [21046]	35% [11335]	100% [32381]

Numbers in brackets represent raw probe responses; percentages represent average proportions across individuals.

### Temporal stability of ratings

To assess whether ratings drifted systematically over the course of the experiment (e.g. due to boredom or fatigue), we modeled probe position as a continuous predictor of freeness and directedness ratings using linear mixed-effects models with random intercepts and random slopes for probe position by participant. Both dimensions showed statistically detectable but negligible linear drifts (freeness: β = −0.0035, *SE* = 0.0012, *t* = −2.92, *P* = .004; directedness: β = −0.0051, *SE* = 0.0014, *t* = −3.64, *P* < .001), corresponding to changes of less than 0.35 scale points across the entire experiment, indicating no meaningful temporal confound.

### Qualitative analysis of participant explanations (Experiment 4)

Two independent raters read 6 316 qualitative responses. The central question of this qualitative analysis was not whether explanations could be sorted into freely moving versus deliberately directed categories—the quantitative ratings already captured that distinction—but rather how participants arrived at whatever ratings they gave: did they rely on a direct phenomenological sense of how their thoughts were moving (dynamic-related), or did they arrive at their ratings through other means, such as inferring thought dynamics from noticing changes in thought content (content-related)? From the 6316 explanations, the key finding was that a majority (~60%, *n* = 3790) contained explicit dynamic-related words, suggesting reliance on the felt phenomenology of thought dynamics (see Table 2 for a summary of category frequencies). The remaining 40% (2526) did not contain explicit dynamic-related language. These participants may have relied on content-based inferences, though it is also possible that they experienced thought dynamics directly but did not spontaneously use dynamic-related words (e.g. due to time constraints, the salience of content, or an assumption that content descriptions were sufficient; see also Proust 2010, Carruthers 2009).

**Table 2:** Summary of qualitative analysis (Experiment 4, *N* = 542).

**Category**	***N***	**% of explanations**	**Directed (%)**	**Freely moving (%)**
Dynamic-related language	3 790	60	N/A	N/A
Content-related inference	2 526	40	N/A	N/A
*Additional categories*				
Mind-blanking	323	5	17	60
Perceptual coupling	631	10	25	75
Experiment-related	316	5	36	64
Meditation	38	0.6	—	—

Full multilevel model results for meditation and experiment-related categories are reported in Appendix D.3. Directed/Freely moving columns show proportions within each category. Note: Percentages are raw descriptive proportions; model-estimated probabilities reported in the text may differ due to the multilevel structure of the data. Percentages are not reported for categories with very small sample sizes. The dynamic versus content categories reflect how participants justified their ratings rather than the ratings themselves, and therefore are not split by directedness or freeness.

Because the dynamic versus content coding addressed how participants justified their ratings rather than the ratings themselves, these categories were analyzed descriptively without splitting by directedness or freeness. In contrast, the analyses below examined whether particular experiential contexts (e.g. mind-blanking, perceptual coupling) showed systematic imbalances in directedness or freeness ratings. For each category, we analyzed the subset of probes containing that category and tested whether directedness or freeness responses occurred more frequently than their alternatives using mixed-effects logistic regression models with random intercepts for participants.

We found that only 5% of the rationales reflected zoning out/mind-blanking, or a similar state. Mind-blanking episodes were significantly more likely to be classified as not directed than directed (β = −1.99, *SE* = 0.34, *z* = −5.93, *P* < .001), corresponding to an estimated probability of directed responses of 0.16. Freely moving responses were also more common than not freely moving responses (β = 0.85, *SE* = 0.27, *z* = 3.18, *P* = .001), corresponding to a probability of freely moving responses of 0.70.

Meditation episodes were rare (0.6%; 38 probes across 22 participants) and almost exclusively classified as freely moving and not directed (Appendix D.3). Experiment-related thoughts occurred in ~5% of all trials (299 probes across 165 participants). These showed a similar pattern, with freely moving responses more common than directed responses (Appendix D.3).

Regarding perceptual (de)coupling, 10% of explanations contained explicit perceptually-coupled words. Perceptually coupled episodes were significantly more likely to be classified as not directed than directed (β = −1.20, *SE* = 0.13, *z* = −9.40, *P* < .001), corresponding to an estimated probability of directed responses of 0.23. Freely moving responses were also highly prevalent during perceptually coupled episodes (β = 1.50, *SE* = 0.20, *z* = 7.67, *P* < .001), corresponding to a probability of freely moving responses of 0.82. Decoupled episodes showed a similar but less extreme pattern, with directed responses occurring with a probability of 0.28 (β = −0.93, *SE* = 0.06, *z* = −14.71, *P* < .001) and freely moving responses occurring with a probability of 0.71 (β = 0.89, *SE* = 0.07, *z* = 13.21, *P* < .001). Direct comparisons confirmed that decoupled episodes were more likely to be classified as directed (β = 0.43, *SE* = 0.11, *z* = 3.85, *P* < .001) and less likely to be classified as freely moving (β = −0.53, *SE* = 0.11, *z* = −4.71, *P* < .001) than perceptually coupled episodes.

## Discussion

Our findings suggest that the stream of thought can be characterized by reference to the dynamics at play between mental states. Specifically, freely moving and deliberately directed thoughts can be characterized as two distinguishable dynamic dimensions, distinct from thought content in the probed resting-state stream of thought. Thoughts during such a resting state were approximately twice as likely to be freely moving as directed. Although freely moving and deliberately directed thoughts showed a negative correlation, our results suggest that the two dimensions remain conceptually and empirically distinct. The negative correlation is consistent within the majority of individuals and across contexts—laboratory-based, online, and inside the fMRI scanner.

The negative correlation (*ρ* = −0.43 across contexts) we found in the present study between these dynamics indicates that, while the two dimensions share variance, they are far from redundant as only ~18% of the variance is shared between the two dimensions. This suggests that participants were able to differentiate between them in real time despite their apparent conceptual opposition. This partial dissociation aligns with prior research suggesting that thought dynamics may coexist or shift fluidly within a single thought stream (e.g. Kam et al. 2021). This finding is consistent with the DFT framework, which posits that freely moving thoughts arise when there are relatively few constraints on the transition between mental states, whether deliberate or automatic, and that these dynamics are not bipolar opposites but rather may influence each other across different contexts (Christoff et al. 2016, Girn et al. 2020).

We found that participants may have relied on two complementary metacognitive strategies when rating the freeness and directedness of their thoughts. In ~60% of explanations, participants referenced the felt, experiential quality of their thought dynamics directly. In the remaining 40%, participants did not use explicit dynamic-related words, which could reflect content-based inference, insufficient time, the salience of content, or an assumption that content descriptions were sufficient (Koriat 1993, Schooler 2002).

In addition, mind-blanking, meditating, and feeling constrained by the experimental setup were not significant sources of interference in our study. Finally, we found that the dimension of perceptual (de)coupling, commonly studied in mind-wandering studies, was partially dissociable from the two dynamic dimensions in our study.

Prior work has shown that content-based dimensions (such as task-relatedness) are independent of dynamic dimensions (such as freeness and directedness): Mills et al. (2018) found task-relatedness was independent of free movement; O’Neill et al. (2021) found that off-task thought rates fluctuated with task demands but freely moving thought did not; Smith et al. (2018) showed these dimensions had different circadian patterns; and Kam et al. (2021) found that task relatedness explained only 26% of the variance in dynamic categories including freely moving and deliberately directed thought (see also Smith et al. 2022). Our findings extend this work by showing the dissociability of freely moving and deliberately directed thoughts in a probed resting-state context.

The negative correlation between freeness and directedness may partly reflect the antithetical framing of the definitions provided to participants (e.g. ‘no overarching purpose’ versus ‘deliberate purpose’). Participants were instructed to rate the extent to which each description characterized their thoughts, not that all criteria had to be met. However, the overlapping antithetical language likely contributed to the observed correlation. Future studies could test this by using definitions that minimize overlapping criteria. Despite this limitation, if the negative correlation were purely an artifact of definitional overlap, we would expect all participants to show correlations near −1. Instead, 19% of participants showed zero or positive correlations, and the full range of intra-individual correlations spanned from −1 to +1 (Appendix D.2). This substantial individual variability suggests that participants were reporting genuine phenomenological differences rather than merely following the logical structure of the definitions.

Our study relied on single-item measures of each construct. Although single items minimize probe duration in experience-sampling paradigms, they do not allow assessment of within-item reliability, and there is no strong reason to believe that all participants interpret these items identically or use them consistently across time. Future studies could incorporate multiple items per construct and constrained scaling techniques (West et al. 2000, Ward et al. 2015) to standardize rating-scale usage.

Sripada and Taxali (2020) showed that a stream of thought has a clump-and-jump pattern in terms of how closely related adjacent mental states are in terms of content. We are yet to fully understand how these patterns relate to thought dynamics. Additionally, specific types of phenomenology pertaining to the dynamics of thought can be explored in future studies. For example, Stan and Christoff (2018) discussed the ‘easeful’ way in which thoughts move over time as a defining characteristic of spontaneous thought.

Another observation from the present study was that ~5% of the qualitative responses were reflective of mind blanking or zoning out. This is consistent with previous work that showed a similar frequency of mind blanking during an experiment (Andrews-Hanna et al. 2010). Although the various terms classified as mind-blanking in our study could be indicative of distinct experiential states, a common thread is that they seem to suggest being either devoid of content or, at least, devoid of access to thought content. In this study, participants provided corresponding ratings regarding their thought dynamics, even while describing their mental state as blank. It would be interesting to explore whether the state of mind-blanking continues to retain access to thought dynamics, even when it loses access to thought content. If this were the case, mind blanking could be a possible medium to delineate thought dynamics as distinct from thought content. Alternatively, future studies could accommodate such a state by providing a corresponding option on the rating scale, as a recent fMRI study showed that mind-blanking has a distinctive neurobehavioral profile with a unique neural composition (Mortaheb et al. 2022), and meditation and mind-blanking may reflect distinct mental states (Fox et al. 2012). Mind blanking may offer a useful contrast case for distinguishing thought dynamics from thought content, although this remains a hypothesis for future work rather than a conclusion from our data, given its low prevalence in our study.

We also examined perceptual (de)coupling, a dimension commonly studied in mind-wandering research. Independent of the ratings of freeness and directedness, the majority of rationales (~90%) reflected perceptually decoupled thoughts, whereas only ~10% reflected perceptually coupled thoughts, although some rationales may have reflected aspects of both. Notably, ~75% of perceptually coupled thoughts were rated as freely moving. This pattern indicates that perceptual coupling is not equivalent to directedness and that freely moving thought dynamics can also occur during externally oriented cognition. More broadly, these findings suggest that the dynamics governing how thoughts unfold are not tightly determined by whether attention is coupled to the external environment. Instead, perceptual coupling and the phenomenology of thought movement appear to represent partially independent dimensions of experience. Given that attention continually shifts between internal and external environments (Chun et al. 2011, Dixon et al. 2014), this independence is perhaps unsurprising. These results are consistent with the Dynamic Framework of Thought, which distinguishes the dynamics of thought from other dimensions such as task relatedness or perceptual decoupling (Christoff et al. 2016). Because participant explanations were typically brief and sometimes contained multiple experiential aspects simultaneously, the coding scheme intentionally used relatively broad categories rather than attempting fine-grained distinctions between perceptual states e.g. instances of overlap between perceptual coupling and decoupling.

Whereas the present study primarily concentrated on analyzing the broad categories and extent of constraints within thoughts, there remains a rich avenue for future research to examine constraints more closely. For instance, future investigations could examine varying levels of constraint, such as the degree of goal abstraction, within intentionally directed thought processes (Christoff et al. 2009b, Klinger 2013, Dixon et al. 2014, Zamani et al. 2022). It is worth noting that, albeit infrequently, participants occasionally described their thoughts as freely moving and deliberately directed (Table 1). In many of these instances, qualitative responses suggested that such thought patterns encompassed both deliberately directed aspects (e.g. focused on an essay topic) and freely moving (e.g. loosely constrained ideas evolving over time).

Studies on large-scale neural networks have proposed various mechanisms for cognitive control operating on different timescales. The frontoparietal control network appears to play a role in modulating short-term cognitive control, whereas the cingulo-opercular control network may be involved in tasks with abstract and long-term goals (Dosenbach et al. 2007). Some studies have described constrained thought as the opposite of free movement (Alperin et al. 2021, O’Neill et al. 2021). The present study indicates that this is not necessarily the case, as the two were not perfectly anticorrelated (i.e. low free movement ≠ highly constrained) in our study. However, it is also important to note that the category of constrained thought refers to thoughts that can be constrained in two distinct ways: deliberate, via cognitive control, and automatic (Christoff et al. 2016). The latter type of constraint describes thoughts that appear to draw or pull attention and seem to operate outside the mechanisms of cognitive control. Rumination and obsessive thoughts are examples of thoughts that are highly automatically constrained but with weak deliberate constraints (Christoff et al. 2016, DuPre and Spreng 2018). According to Andrews-Hanna et al. (2022), people who ruminate more are drawn to negative conceptual environments and are more likely to stay there for longer. However, the relationship between deliberately constrained, freely moving, and automatically constrained thought remains incompletely understood.

Although phenomenological reports have inherent limitations, converging evidence supports the meaningfulness of participants’ ratings: (i) the pattern was consistent across three experimental contexts; (ii) response distributions remained stable throughout extended sessions; and (iii) the diversity of qualitative responses suggests participants were not simply echoing instructional language. The present study, however, did have several additional limitations. The probed resting-state paradigm, while useful for minimizing external task demands, does not simulate real-world conditions. The experiment duration (60–90 minutes) was long, and some participants reported boredom or fatigue, although our results show that drift effects in probe ratings were negligible. Additionally, the fixed question order (freeness always preceding directedness) may have introduced anchoring effects; future studies could counterbalance question order to rule this out. The codebook for classifying qualitative explanations was generated from the same dataset to which it was applied; while this is less susceptible to overfitting than machine-learning methods—because the codebook consisted of individual words (e.g. ‘flowing,’ ‘stuck’) whose dynamic meaning is semantically transparent and was judged by human coders independently of statistical patterns in the data—future studies could nonetheless use a split-sample approach to further strengthen generalizability.

Thought dynamics also have clinical relevance: maladaptive rumination can be characterized by both content and dynamics (Raffaelli et al. 2021), and people with ADHD engage in a higher proportion of freely moving off-task thoughts than non-ADHD controls (Alperin et al. 2021). Thought dynamics may also have therapeutic significance, such as promoting insight by relaxing constraints on thought (Stan and Christoff 2018).

The present study provides an initial exploration of how people experience and differentiate thought dynamics through the use of introspective methods. These findings are consistent with those of Sheth et al. (2025) who found that distinct brain networks are associated with freeness *versus* directedness. Our findings open avenues for future neurophenomenological research, where first-person reports of thought dynamics can be further linked to third-person data (Lutz and Thompson 2003, Thompson et al. 2005, Northoff and Heinzel 2006, Christoff et al. 2009a, Kühn et al. 2014, Gonzalez-Castillo et al. 2021, Kam et al. 2021, Smallwood et al. 2021, Sheth et al. 2025).

## Supplementary Material

DOT-ES_Revised_Appendices_niag017

## Data Availability

The datasets used and/or analyzed during the current study are available from the corresponding author upon reasonable request.
