# Supplementary material for: Phenomenology of the stream of thought: dissociable dynamic dimensions revealed through experience sampling
Source: Neurosci Conscious. 2026 May 18;2026(1):niag017. doi: 10.1093/nc/niag017 (PMC13181415; doi:10.1093/nc/niag017)
Supplement: DOT-ES_Revised_Appendices_niag017 [file dot-es_revised_appendices_niag017.docx]

# Appendix A: Experience Sampling Questions

Below is a description of Freely-moving and Directed thought dynamics as shown to participants:

*What do we mean by ‘thoughts’?*

By thoughts we mean anything on your mind, including:

- memories, emotions, and imaginings, as well as
- sensations such as things that you see, hear, smell and so on.

*Question 1: Was your mind moving about freely?*

*(1=not at all to 6=very much)*

Your thoughts move freely when:

- they seem to wander around, flowing from one thing to another
- there is no overarching purpose or direction to your thinking (although there may still be some connection between one thought and the next)
- images and memories seem to spontaneously come into your mind
- your attention lands spontaneously on things in your environment
- your mind may spontaneously drift between things in the external environment and internal images
- it feels like your thoughts could land on pretty much anything
- your thoughts seem to flow with ease

*Question 2: Were you actively directing your thoughts? (1=not at all to 6=very much)*

You actively direct your thoughts when:

- There is a deliberate purpose to your thinking
- You steer your thoughts in a direction of your choosing
- You watch out for thoughts that distract you from your goal and bring your mind back
- You are aware of whether your thoughts serve your goal, and favour those that are most helpful

**Appendix B: Trial Structure**

*
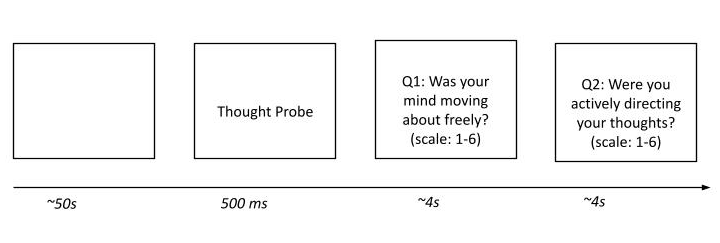
*

**Figure B1.** *Representation of events in a single trial*

A blank screen was presented for approximately 50 seconds. When participants were probed, they were asked to provide their ratings on a scale of 1 to 6 on the two questions.

**Appendix C: Examples of Qualitative Responses for Various Categories**

**C.1. Dynamics-based explanations**

*“There was nothing directing my thoughts and things seem to just appear in my head. My thoughts were transitioning well and I was not really thinking of something in particular.”*

*“My mind was fully able to be free and uncontrolled and I did not direct any of these thoughts and was able to be free in thoug”*

**C.2. Content-based explanations**

*“I saw a pair of scissors that cut papers into cool shapes and then past memories came up of when I would use them for school projects”*

*“i was thinking about christmas and how much money i am going to have to spent while not working during Christmas break. it is quite stressful”*

**C.3. Mind-blanking related explanations**

*“my mind still feels blank. There are no thoughts and everything seems quiet in my mind. like its shut off in a sense”*

*“not thinking about anything just zoning out and blanked”*

**C.4. Perceptually-coupled explanations**

*“i was seeing the precarious picture on the blackboard and wondering what is that, who draw that”*

*“I heard sounds from my neighbour and ended up thinking about it and getit”*

**C.5. Perceptually-decoupled explanations**

*“I wandered into a memory that I dont think was guided by anything around me and was not paying attention to thought direction”*

*“Rehearsing my speech for the event after this study.”*

**C.6. Meditation-related explanations**

*“I was in a meditative state, watching my thoughts move freely but being aware of them and sometimes redirecting”*

*“i gave them these ratings because i was meditating with a clear mind during the time”*

**C.7. Task-related explanations**

*“I am still trying to figure out the point of this experimen”*

*“I was thinking too much about thinking about the experiment and what to write here and its moving too fast the experiment can’t type enough I got stressed out”*

**Appendix D: Qualitative Coding Category Definitions, Supplementary Figure, and Full Model Results**

**D.1. Qualitative coding category definitions**

Explanations were coded using a custom R routine according to the following a priori categories, derived from the inductive–deductive procedure described in the Methods:

a. **Dynamic-related words:** Explanations that contained words implying a sense of movement across time (e.g., flowing, moving, drifting, swiftly) or a lack of movement (e.g., stuck, fixated). Explanations that did *not* include explicit dynamic-related words were classified as content-related.

b. **Mind-blanking:** Explanations that contained words implying that the participant did not have access to the content of their thoughts (e.g., zoning out, blanking out, no thoughts).

c. **Perceptual coupling:** Explanations that contained words implying that the participant was thinking about things related to the external sensory environment (e.g., watching, smelling, listening). This was contrasted with perceptually decoupled explanations that contained words that were either not perceptually coupled (i.e., no explicit reference to the external or internal environment) or explicitly perceptually decoupled words that referred to the internally oriented environment (e.g., feeling, thinking, remembering). Some explanations classified as perceptually coupled reflected a dynamic shift from a state of perceptual decoupling. Our study, however, was not designed to account for these mixed explanations.

d. **Experiment-related:** Explanations that contained words implying that the participant was thinking about things related to the current experimental context (e.g., experiment, study, probe).

e. **Meditation:** Explanations that contained words implying that the participant was meditating (e.g., meditating, mindfulness).

**D.2. Distribution of intra-individual correlations**

To complement the violin plots presented in the main text (Figure 2), the histogram below shows the full distribution of intra-individual Spearman correlations between freeness and directedness across all 708 participants. Whereas 81% of participants showed a negative correlation (–1 ≤ ρ < –0.1), 19% showed correlations near zero or positive (–0.1 ≤ ρ ≤ 1).


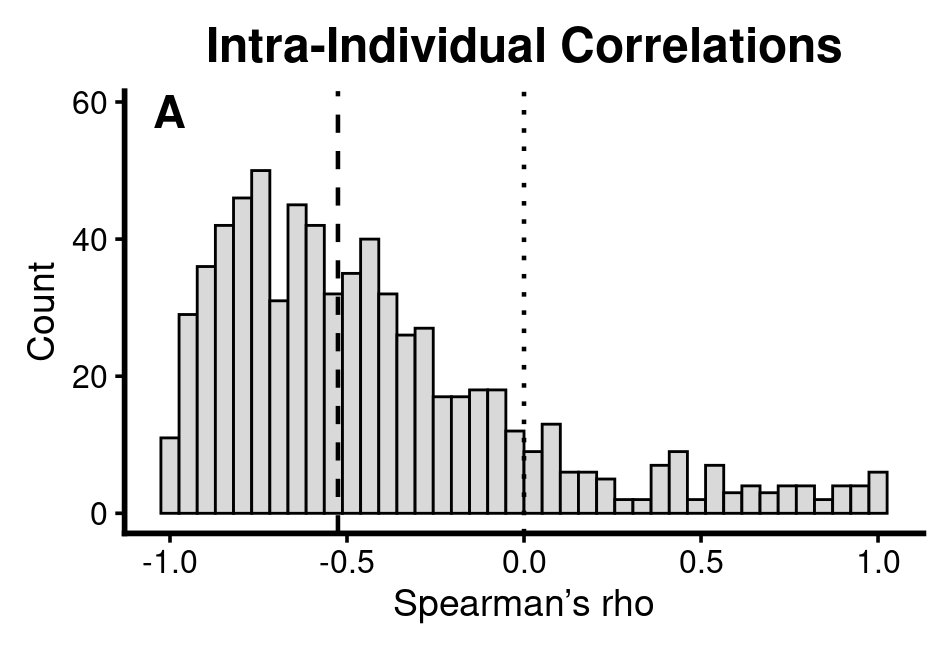


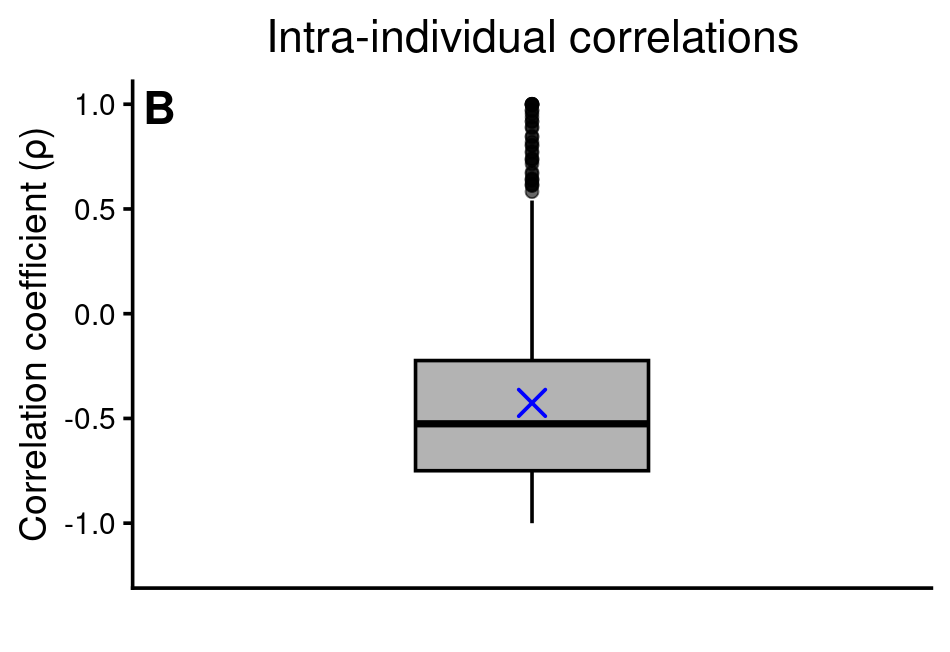


**Figure D1.** *Correlation between freeness and directedness across 708 participants. (a) Histogram of intra-individual correlations. (b) Box-Whisker Plot from intra-individual correlations (Median = –0.53, Mean = –0.43, SD = 0.44).*

**D.3. Full multilevel model results for meditation and experiment-related categories**

The main text summarizes meditation and experiment-related thought categories briefly; full multilevel logistic regression results are reported below.

***Meditation***

Meditation episodes were rare (0.6% of all probes; 38 probes across 22 participants) but showed a highly consistent pattern of responses. Descriptively, meditation episodes were almost never classified as directed and almost always classified as freely moving. To account for repeated probes within participants, we fitted mixed-effects logistic regression models including participant as a random intercept. These models confirmed that meditation episodes were significantly more likely to be classified as not directed than directed (β = −9.94, SE = 3.50, z = −2.85, p = .004), and significantly more likely to be classified as freely moving than not freely moving (β = 9.86, SE = 3.48, z = 2.83, p = .005). A direct comparison between scales further showed that the probability of a positive response differed significantly between freeness and directedness (β = 4.93, SE = 1.25, z = 3.96, p < .001). However, because meditation probes were rare and responses were highly consistent, these estimates should be interpreted cautiously.

***Experiment-related thoughts***

Experiment-related thoughts occurred in about 5% of all trials (299 probes across 165 participants) and showed a consistent pattern of responses. To account for repeated probes within participants, we fitted mixed-effects logistic regression models including participant as a random intercept. Experiment-related thoughts were significantly more likely to be classified as not directed than directed (β = −0.57, SE = 0.16, z = −3.50, p < .001), corresponding to an estimated probability of directed responses of 0.36. In contrast, freely moving responses were more common than not freely moving responses (β = 0.59, SE = 0.17, z = 3.37, p < .001), corresponding to a probability of freely moving responses of 0.64. A direct comparison between scales further showed that the probability of a positive response differed significantly between freeness and directedness (β = 0.98, SE = 0.17, z = 5.83, p < .001), indicating that positive responses were more likely on the freeness scale than on the directedness scale.
